# Effect of Betulin Colloidal Particles on Proliferation and Cytokine Secretion of Human Skin Fibroblasts

**DOI:** 10.3390/plants12173088

**Published:** 2023-08-28

**Authors:** Una Riekstina, Sanita Vitolina, Karina Goluba, Kaspars Jekabsons, Ruta Muceniece, Rudolfs Berzins, Janis Rizhikovs, Daniela Godina, Arturs Teresko, Aigars Paze

**Affiliations:** 1 Faculty of Medicine, University of Latvia, LV-1004 Riga, Latvia; una.riekstina@lu.lv (U.R.); karina.goluba@lu.lv (K.G.); kaspars.jekabsons@lu.lv (K.J.); ruta.muceniece@lu.lv (R.M.); 2 Biorefinery Laboratory, Latvian State Institute of Wood Chemistry, LV-1006 Riga, Latvia; sanita.vitolina@kki.lv (S.V.); rudis.berzins@gmail.com (R.B.); janis.rizikovs@kki.lv (J.R.); daniela.godina@kki.lv (D.G.); 3 ZS DOKTUS, LV-4101 Cesis, Latvia; arturs_teresko@inbox.lv

**Keywords:** betulin, colloidal particles, gas chromatography, dynamic light scattering, in vitro, human skin fibroblasts, cytotoxicity, cytokine secretion, wound healing

## Abstract

The aim of the study was to obtain untreated and treated betulin colloidal particles and assess their effect on the viability, morphology, proliferation and cytokine secretion of human dermal fibroblasts. To improve bioavailability, betulin treatment was performed by an antisolvent precipitation technique. The average particle size after treatment in the aqueous dispersion decreased from 552.9 ± 11.3 to 278.2 ± 1.6 nm. Treated betulin colloidal particles showed no cytotoxicity up to a concentration of 400 µg·mL^−1^ in the colorimetric tetrazolium salt viability test (CCK-8). Moreover, the cell morphology was not changed in the presence of betulin colloidal particles at a concentration range from 0.78 to 400 µg·mL^−1^. The obtained results also show that betulin particles induce the secretion of the proinflammatory and angiogenesis-stimulating cytokine IL-8. However, further studies would be required to clarify the mechanism of IL-8 secretion induction.

## 1. Introduction

Extracts from the bark and leaves of the *Betula* L. species have displayed a wide range of in vitro and in vivo pharmacological activity, including immunomodulatory, anti-inflammatory, antimicrobial, antiviral, antioxidant, antidiabetic, dermatological, gastroprotective and hepatoprotective activity [1]. Betulin is a major constituent of the lupane-type pentacyclic triterpenes found in birch outer bark and numerous studies suggest that betulin possesses diverse beneficial effects in various diseases, including wound healing [2]. The oleogel formulation of the betulin has been tested in several clinical trials for skin wound treatment. A topical betulin gel consisting of 10% birch outer bark extract and 90% sunflower oil significantly accelerated re-epithelialization and wound closure in a randomized phase III clinical trial [3]. Furthermore, it was demonstrated that Oleogel-S10 improved the outcome of superficial partial thickness burn wounds in a phase III trial and 12 months follow-up [4].

The physiological process of reparative wound healing consists of four stages: hemostasis, inflammatory, proliferation and remodelling [5]. The two main structural layers that form the skin are the epidermis and the dermis, and both human skin keratinocytes and fibroblasts are frequently used as models to demonstrate the wound healing properties in vitro [6]. Birch outer bark dry extract and betulin are thought to work by temporarily upregulating proinflammatory modulators like cyclooxygenase 2, interleukin IL-6 and IL-8 in primary human keratinocytes cells and dermal fibroblasts [7,8,9]. Numerous studies also suggest that betulin may be helpful for tissue regeneration and skin renewal. It has been shown that betulin and its derivative betulin ester with diaminobutyl acid increased the production of type I and type III collagen in human gingival fibroblasts [10]. The tissue regeneration mechanism of betulin is based on the inhibition of inflammatory responses, wound pigmentation and downregulation of pro-apoptotic factors like Caspase3 as demonstrated in a zebrafish in vivo model [11].

Its limited water solubility has been one of the main challenges for betulin formulations for topical medicinal uses. The most recent method for increasing wettability is to create betulin nanoparticles with smaller sizes and greater particle surface areas. Water-in-oil foams and oleogels contain betulin particles with an average size of 5800 nm or larger [12]. It has been suggested that particles smaller than 200 nm could be taken up by endocytosis and exert biological activity on cells [13]. Purified betulin nanoparticles can be obtained by precipitation from an ethanol solution with an aqueous antisolvent technique. The nanoparticle production process was optimized and the concentration of betulin in ethanol 6 mg/mL at a solution temperature of 40 °C resulted in an average particle size of 110 nm. The results from in vivo studies in rat models showed that betulin nanoparticles have 1.21 times higher bioavailability than untreated betulin raw material [14]. A hydrogel that supports cell proliferation, migration, differentiation and blood vessel creation could be used for wound healing purposes [15].

Considering that betulin colloidal particles from birch outer bark could function as an ecological gellant and emulsifier, makes it highly promising for further use in the cosmetic industry. Human skin fibroblasts produce and remodel an extracellular matrix as well as communicate with other skin cells via secretory factors in normal physiological conditions and during the wound healing process [16]. The aim of this study was to obtain untreated and treated betulin colloidal particles and assess their effect on the viability, morphology, proliferation and cytokine secretion of human dermal fibroblasts.

## 2. Results

### 2.1. Characterization of Betulin and Its Aqueous Dispersions

**Figure 1:**
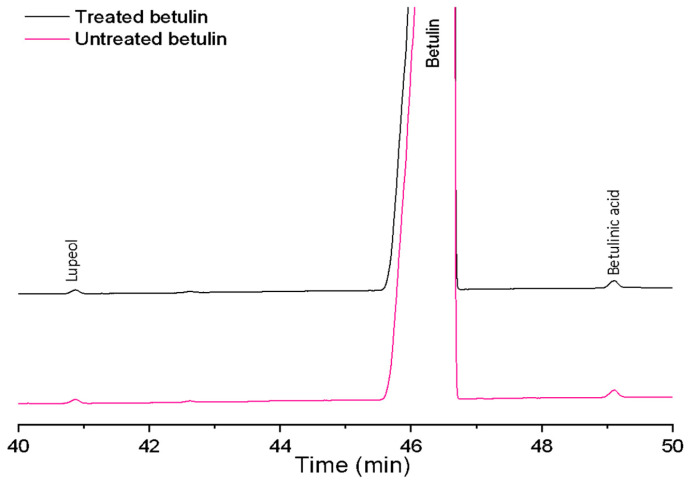
GC-FID chromatograms of treated and untreated betulin samples.

Characteristics of basic composition of the dry untreated and treated betulin samples listed in Table 1 show some minor differences, indicating that an additional purification process occurred during the preparation of the betulin colloidal particles by liquid antisolvent precipitation. Partial purification (loss of dry matter) probably occurs because some components of betulin powder are soluble in water [17] and remain in the filtrate. The gas chromatography analysis (Figure 1) shows that the betulin content increased from 97 to 99 percentage by weight (wt%), while the impurity of phenolic compounds measured spectrophotometrically decreased from 0.28 to 0.21 wt%.

**Figure 2:**
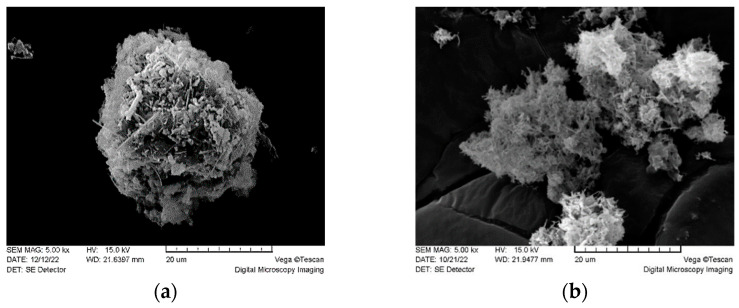
SEM micrographs of untreated betulin powder sample (**a**) and treated betulin powder sample from lyophilized hydrogel (**b**).

The bulk densities of each dry betulin powder were determined according to the USP method [18] in triplicate. The results showed that bulk density of untreated betulin powder was significantly larger than the treated, 1460 ± 21 and 408 ± 22 mg·mL^−1^, respectively. Scanning electron microscopy images in Figure 2 confirm the morphological changes in the particles after treatment, resulting in a more uniform particle shape and a decrease in the density of the particles in their agglomerates.

**Figure 3:**
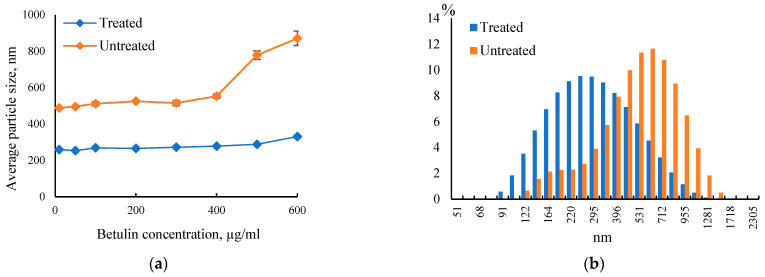
The effect of betulin concentration on average particle size in aqueous dispersion (**a**) and histograms of betulin particle size distribution at a concentration of 400 µg·mL^−1^ (**b**).

The size of the betulin particles in aqueous dispersion was measured using dynamic light scattering technique. To avoid concentration dependent effects, the influence of the concentration of betulin particles in dispersion on the average particle size was investigated in the concentration range of 10–600 µg·mL^−1^ (Figure 3a). It can be seen that the average particle size of treated betulin in aqueous dispersion is in principle independent of concentration over the concentration range studied. However, a sharp increase in average particle size can be observed for the untreated sample as the particle concentration increases above 400 µg·mL^−1^. Based on the results obtained, and for further studies on human dermal fibroblasts, a maximum betulin concentration of 400 µg·mL^−1^ in the dispersion was chosen to exclude the possibility of particle agglomeration. Particle size distributions for both betulin samples at concentration 400 µg·mL^−1^ are shown in Figure 3b. The treated betulin sample exhibited significantly smaller average particle size of around 278.2 ± 1.6 nm with lower polydispersity index of 0.249 ± 0.018 than the untreated one (552.9 ±11.3 nm; PDI 0.387 ± 0.023).

### 2.2. The Effect of Betulin Dispersions on Cell Viability and Morphology

**Figure 4:**
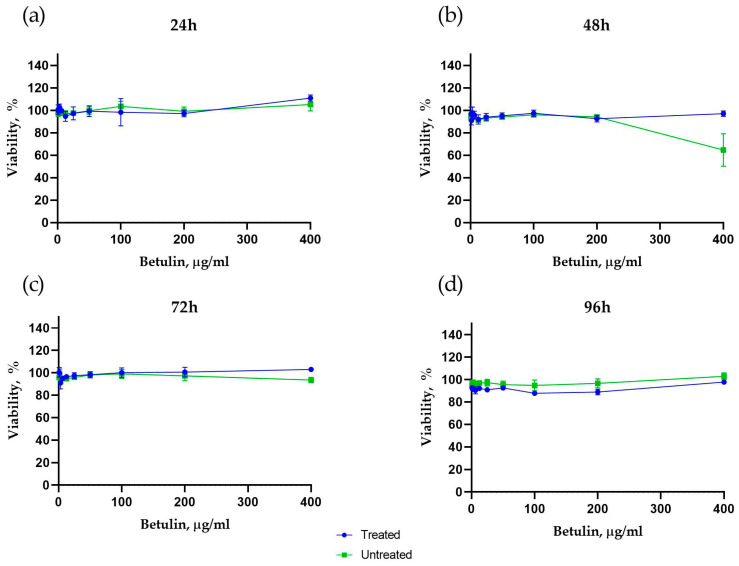
The effect of treated and untreated betulin particle dispersions on the viability of human dermal fibroblasts after 24 h (**a**), 48 h (**b**), 72 h (**c**) and 96 h (**d**) incubation.

The effect of betulin dispersions on cell viability was analysed using a colorimetric tetrazolium salt-based CCK-8 assay. Cytotoxicity assay results show that untreated and treated betulin did not affect cell viability after 24 h incubation (Figure 4a), whereas after 48 h, the viability decreases to 60% at the 400 µg·mL^−1^ concentration of betulin dispersion (Figure 4b). The effect of this particular concentration of untreated betulin dispersion is transient because at the 72 and 96 h timepoints the viability is more than 90% (Figure 4c,d). Noteworthy, after 96 h incubation, the viability of cells incubated with treated betulin colloidal particles is on average 10% lower compared to untreated betulin (Figure 4d).

**Figure 5:**
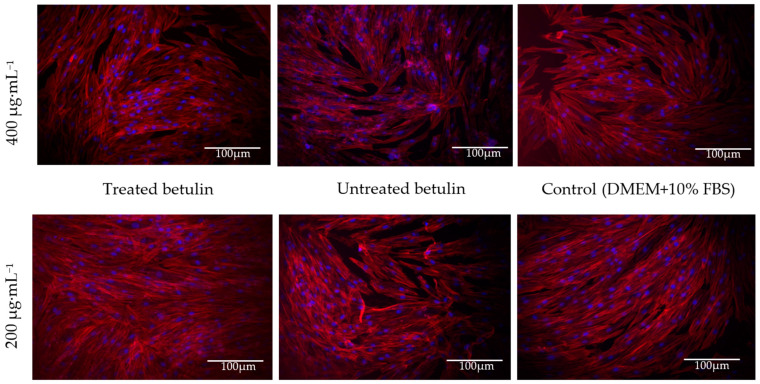
The morphology of human dermal fibroblasts after 96 h incubation with untreated and treated betulin at 200 µg·mL^−1^ and 400 µg·mL^−1^. Red colour—actin labelled with Actin Red 555, and blue colour—Hoechst-labelled nuclei. Representative images are shown.

In line with the cell viability assay, the cell morphology analysis by fluorescence microscopy showed no effect of betulin dispersions on human dermal fibroblast morphology. There are no visible changes observed following cell incubation with treated and untreated betulin at concentrations up to 400 µg·mL^−1^ at different time points. The cells possess a characteristic spindle-like fibroblast shape with centrally placed oval nuclei, and the confluence is near 100% (Figure 5).

### 2.3. The Betulin Colloidal Particle Effect on Cytokine IL-8 and IL-1b Secretion

**Figure 6:**
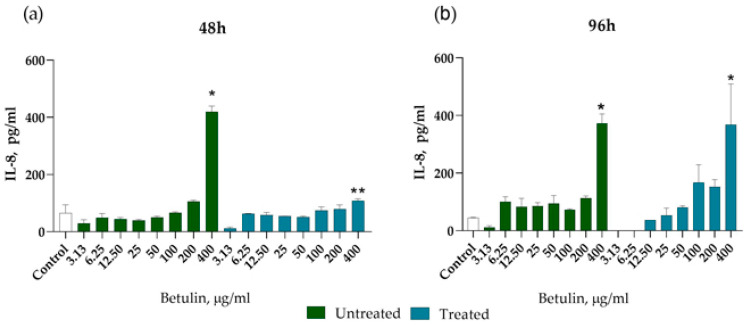
Treated and untreated betulin induced IL-8 secretion from dermal fibroblast cultures after 48 h incubation (**a**) and 96 h incubation (**b**). * *p* < 0.05 compared to control, ** *p* < 0.05 when comparing substances at a concentration of 400 µg·mL^−1^ (Anova test, Holm–Sidak post-test).

We used the ELISA method to evaluate the effect of betulin colloidal particle dispersions on IL-8 and IL-1b secretion. After 48 h incubation with untreated betulin dispersion, an increase in IL-8 secretion can be observed at concentrations of 200 µg·mL^−1^ and 400 µg·mL^−1^, and it is statistically significantly different from the secretion level of control cells (Figure 6). Results show a fourfold increase in IL-8 secretion (280 pg·mL^−1^) in cells incubated with 400 µg·mL^−1^ untreated betulin, while in case of treated betulin there is a twofold increase (140 pg·mL^−1^) in the IL-8 level, compared to control cells (70 pg·mL^−1^) (Figure 6a). After 96 h, the IL-8 level increased to 370 pg·mL^−1^ (Figure 6b) in cells incubated with untreated betulin and there was a fourfold increase of up to 370 pg·mL^−1^ after incubation with treated betulin colloidal particles (Figure 6b).

Betulin dispersions did not induce IL-1b secretion in dermal fibroblast cultures.

## 3. Discussion

One of the main issues with betulin dispersions for topical applications has been its low-water solubility. The most current technique for improving wettability is to make smaller and more surface-area-rich betulin nanoparticles. To obtain micro- and nanoparticles of birch outer bark betulin extractives, it is advantageous to use an antisolvent precipitation technique. It is generally a simple and cost-effective approach with good scaling potential. By determining the maximum solubility of birch outer bark betulin samples at the boiling point of ethanol and then pouring a saturated solution into various quantities of distilled water, a more effective technique for generating colloidal particles was introduced [19]. The outcome was a dispersion with colloidal particles that has been concentrated and separated via straightforward filtration through filter paper in a Buchner funnel under vacuum. As a result, homogeneous supramolecular hydrogels were obtained, which, depending on the dilution, could absorb a certain amount of liquid. The studies showed that the smaller the average size of the birch outer bark betulin particles in the hydrogels, the more liquid the hydrogel was able to absorb. Under optimal conditions, a hydrogel was obtained from the purified betulin, which had a sorption capacity of 37.45 g of liquid per 1 g of hydrogel dry matter and contained colloidal particles with an average size of 189.8 nm, of which almost 20% was the particle fraction with a size below 140 nm and more than 20% particle fraction with a size between 140 and 190 nm [19]. To obtain dry birch outer bark betulin colloidal particles and to keep the average particle size as low as possible, the best method for gentle drying of hydrogels is lyophilization.

Due to the wide variety of possible uses in pharmacology, cosmetics and the food sector, the betulin micro/nanoparticle hydrogels, in which betulin functions simultaneously as a biologically active substance and as a colloidal surfactant, are of particular interest. It has been reported that the hydrogel formulation is a promising wound dressing due the improved antibacterial and wound healing promoting properties [20]. The literature reports the beneficial effect of sodium alginate-based hydrogel films loaded with *Betula utilis* bark extract on wound closure within an excision wound model in albino rats [21].

Besides animal models, human skin in vitro cell culture models, e.g., keratinocytes and fibroblasts, have been an important tool to study how cells behave and respond to a variety of stimuli, including betulin and its derivatives [22,23]. The main solvent utilised in in vitro studies on betulin is dimethyl sulfoxide (DMSO), which can exhibit significant cytotoxicity on various cell lines at a concentration of 1.25% (*v*/*v*) [24]. Betulin dissolved in DMSO showed cytotoxicity on murine fibroblasts NIH/3T3 at the concentration 0.976 µg·mL^−1^ in the 3-(4,5-dimethylthiazol-2-yl)-2,5-diphenyl-2H-tetrazolium bromide (MTT) test. The IC50 of betulin after 48 h incubation with human skin fibroblasts (WS1) was 3.58 µM (approximately 1.6 µg·mL^−1^) [25].

In the current study, we observed that treated betulin colloidal particles showed no cytotoxicity up to a concentration of 400 µg·mL^−1^ in the colorimetric tetrazolium salt viability test (CCK-8). Moreover, the cell morphology was not changed in the presence of betulin colloidal particles at a concentration range from 0.78 to 400 µg·mL^−1^.

Proinflammatory cytokines including IL-6, IL-8, and tumour growth factors beta-1 and beta-2 (TGF-1, TGF-2) that are generated by fibroblasts and macrophages have a role in mediating the inflammatory response. A recent study showed that the fibroblasts are the best responder cells to the epidermal damage, as demonstrated by the increased IL-8 secretion 48 h after exposure to the keratinocyte extract [26]. Interestingly, both betulin dispersions induced IL-8 secretion following 48 h incubation with dermal fibroblast cells, which was statistically significant at a concentration of 400 µg·mL^−1^. The effect of treated betulin on IL-8 secretion from dermal cells was most pronounced after 96 h of incubation, starting with a concentration of 100 µg·mL^−1^. Noteworthily, increased IL-8 mRNA expression has been observed in dermal fibroblasts from healthy donors and diabetic patients after 24 h incubation with betulin dimethyl sulfoxide (DMSO) solution at a concentration of 0.87 µg·mL^−1^ [8]. Importantly, chemokine IL-8 (CXCL8) has been associated with neutrophil recruitment in the wound healing proinflammatory stage, and early wound neoangiogenesis in the proliferation stage [27]. Neither betulin nanoparticles induced IL-1β which might indicate that particles themselves do not cause inflammatory response, which is in line with a previous report [8]. Further research is needed to draw conclusions on betulin colloidal particle effect on skin fibroblast function in wound healing.

## 4. Materials and Methods

### 4.1. Raw Material

Isolated birch (*Betula pendula Roth*) outer bark was supplied from a plywood factory in Latvia. It was dried at room temperature (moisture content 4–5 wt%) and milled in an SM 100 cutting mill (Retsch GmbH, Haan, Germany) to pass through a sieve with pores measuring 4 mm in diameter. Milled birch outer bark was fractionated using an AS 200 Basic vibratory sieve shaker (Retsch GmbH, Haan, Germany) and a fraction of 1–3.15 mm was collected.

### 4.2. Betulin Extraction

Unpurified raw betulin extract was obtained by organic solvent extraction using ethanol. On the average, 3 kg of birch outer bark and 18 L of the ethanol were used for the extraction using a 30 L reactor. This extraction was conducted in a 30 L reactor, which was equipped with a steam heating jacket, a mechanical stirrer and a reflux condenser. The mixture was stirred and heated to reach the boiling point of the solvent and then it was boiled and stirred at 80 rpm for 1 h. After that, the solution was filtered hot through a 100 μm filter bag and on average 14 L of solution was collected. Further 14 L of the fresh solvent was added to the reactor and continued the extraction. The resulting mixed solutions were evaporated in a 20 L round bottom flask on a Hei-VAP Industrial vacuum rotary evaporator (Heidolph Instruments GmbH & Co., Schwabach, Germany). The obtained wet paste-like extract was dried in an oven at 80 °C for 24 h. The dry extract agglomerates were crushed in a ball mill and fractionated to pass through a 125 µm sieve.

The recrystallization process with ethanol was used to obtain the purified betulin dry powder from unpurified betulin, as described in our previous study [28]. After recrystallization, the betulin content in the extract increased from 52 to 97%.

### 4.3. Obtaining of Betulin Colloidal Particles

The betulin colloidal particles were obtained by employing a liquid antisolvent precipitation technique. Purified betulin dry powder (untreated betulin) was dissolved at maximum saturation in 0.25 L of ethanol at boiling point using a 1 L glass reactor equipped with an electrical heater, mixer and reflux condenser. Maximum saturation was determined empirically, where a known quantity of extractives was added to the solvent and heat was applied until the extractives were fully dissolved. After dissolution, more extractives were added gradually until the opalescence of the solution was observed. At a temperature of 78.3 °C, a maximum saturation concentration of 22 g·L^−1^ was achieved. After that, the hot, saturated solution with stirring was slowly poured in 1.75 L of water (25 °C). Fine particles of the obtained dispersion in 12.5 vol% dilute ethanol solution were concentrated by filtration in a Buchner funnel under vacuum using a filter paper with 6 μm particle retention. After filtration, betulin colloidal particles in the form of a homogeneous hydrogel with a high moisture content (95 wt%) were obtained. The hydrogel was then lyophilized at −50 °C for 48 h using a Heto PowerDry, PL3000 freeze-dryer (Heto Lab Equipment, Allerod, Denmark) to obtain a dry powder of colloidal particles (treated betulin).

### 4.4. Preparation of Untreated and Treated Betulin Dispersions

A total of 40 mg of untreated and treated betulin samples were dispersed in 100 mL sterilized deionized water with a rotor homogenizer Ultra-Turrax T10 basic (IKA Labortechnik, Staufen, Germany) for 4 min at 11,500 rpm and then with an ultrasonic homogenizer Hielscher UP200Ht (Hielscher Ultrasonics, Teltow, Germany) for 2.5 min at 20% amplitude, 150 W power and 60% continuance. After intermediate cooling for 5 min, homogenization with ultrasonic homogenizer was repeated for another 2.5 min.

Both betulin samples were sterilized by autoclaving before preparation of dispersions. Autoclaving treatment was performed at 121 °C for 20 min using 3870ELV-D autoclave sterilizer (Tuttnauer, Breda, Netherlands).

### 4.5. Gas Chromatography with Flame Ionization Detector (GC-FID) Analysis of Triterpenes

To determine the triterpenoid (betulin, lupeol and betulinic acid) composition of betulin samples, gas chromatography analysis with flame ionization detector was performed using a Shimadzu Nexis GC 2030 apparatus (Shimadzu Corporation, Kyoto, Japan). Betulin samples and analytical standards were dissolved in 250 µL of THF and 250 µL of pyridine. Then, 250 µL of silylating mixture III (N,O-bis(trimethylsilyl)trifluoroacetamide, 1-(trimethylsilyl)imidazole and trimethylchlorosilane in the volume ratio 3:3:2) was added and solutions were heated for 1 h at 70 °C. For the component separation, a Phenomenex Zebron ZB-35 (30 m × 0.25 mm × 0.25 µm) column was used. Injection volume was 1 µL at a temperature of 280 °C in a split mode (1:20). The detector temperature was 330 °C and the hydrogen flow rate was 32 mL min^−1^. Two parallel aliquots of each extract were analysed by triple injections. The mutual variation of average values was less than 5%.

### 4.6. Determination of Phenolic Compounds

Standard solutions of gallic acid (GA) (97.5–102.5% (titration), Merck, Darmstadt, Germany) were prepared in the range from 0.02 to 0.1 mg∙mL^−1^. Dry betulin samples were prepared in methanol as follows—approximately 0.2 g of sample was diluted in 10 mL methanol. Filtrate solutions were analysed without additional dilution. A total of 7.9 mL of deionized water and 500 µL of Folin–Ciocalteu reagent (2 M (with respect to acid), Merck, Darmstadt, Germany) were added to 100 µL of sample solution or GA standard solution and incubated for 8 min in the dark. Then, 1.5 mL of 20 wt% sodium carbonate (ACS reagent, anhydrous, ≥99.5%, Merck, Darmstadt, Germany) solution was added to the mixture, and it was incubated for further 2 h in the dark. Light absorption at a 756 nm wavelength was determined using Lambda 25 UV/VIS Spectrometer (Perkin Elmer, Waltham, MA, USA). Coefficient of variations for the values of three parallel measurements did not exceed 1%.

### 4.7. Particle Size Analysis

Average particle size and size distribution of betulin particles in aqueous dispersion was measured by a dynamic light scattering instrument Zetasizer Nano-ZS (Malvern Instruments, Worcestershire, UK). All measurements were performed at 25 °C in triplicate.

### 4.8. Scanning Electron Microscopy (SEM)

The morphology of the particles was examined using scanning electron microscope VEGA TS 5136 (Tescan R&D Software Group, Brno, The Czech Republic). Dry samples were put on samples holder using conductive double sided adhesive carbon tape. Then samples were coated with gold plasma in a low vacuum in an argon atmosphere using Emitech K550X sputter coater (Emitech Ltd., Ashford, Kent, UK). Images were obtained in a low vacuum (10^−3^ Pa) in a nitrogen gas atmosphere using 15 kV voltage.

### 4.9. Betulin Cytotoxicity Assay

The cytotoxicity of betulin colloidal particle dispersions was evaluated using primary human dermal fibroblast cultures obtained from post-surgery human skin materials following authorized approval from the Life and Medical Sciences Research Ethics Committee, the University of Latvia (issued 13 February 2023) [29]. The cells were propagated in a cultivation medium containing DMEM (Dulbecco’s Modified Eagle Medium) supplemented with 10% of fetal bovine serum (FBS; Sigma-Aldrich, St. Louis, MO, USA) and antibiotics (100 U·mL^−1^ penicillin, 100 µg·mL^−1^ streptomycin (both from Sigma-Aldrich, St. Louis, MO, USA), 2.5 µg/mL of amphotericin B (Gibco, Paisley, UK)). Cells were grown in tissue culture flasks in a humidified chamber at 37 °C with 5% CO_2_ until reaching 80% confluence. Cell morphology was observed using a digital inverted microscope AMG-Evos X1 (AMG, Washington, DC, USA). Cells at passages five to eight were used in the experiments.

The cytotoxicity of betulin colloidal particle dispersions was analysed using colorimetric tetrazolium salt-based Cell Counting Kit 8 (CCK8) (Sigma-Aldrich, St. Louis, MO, USA). First, 5 × 103 cells per well were seeded in 96-well plates (Sarstedt, Nümbrecht, Germany) with 100 μL of cultivation medium per well. On the next day, the untreated betulin and treated betulin dispersions were added to cell culture plate wells at concentrations of 0.78 µg·mL^−1^, 1.56 µg·mL^−1^, 3.12 µg·mL^−1^, 6.25 µg·mL^−1^, 12.5 µg·mL^−1^, 25 µg·mL^−1^, 50 µg·mL^−1^, 100 µg·mL^−1^, 200 µg·mL^−1^ and 400 µg·mL^−1^ for each compound. Experimental control wells contained cells in a cell culture medium only. All samples and control were analysed in triplicates. Cells were incubated with the compounds for 24, 48, 72 and 96 h at 37 °C, 5% CO_2_ in 90% humidity. At the selected time points, 10 µL of CCK8 reagent was added to each well and incubated for an additional 3 h at 37 °C, 5% CO_2_ in 90% humidity. After the incubation, 100 μL of medium from each well was transferred to a fresh 96-well plate and the optical density (OD) was measured at 450 nm on Tecan Infinite M200 Pro microplate reader using Magellan 7.1 SP1 software (Tecan Trading AG, Männedorf, Switzerland). Relative percent viability was calculated by subtracting the OD of the cell sample treated with betulin colloidal particles from the control cell sample (100% viability). Data were analysed in Microsoft Excel and GraphPad Prism software (Graph Pad Inc., San Diego, CA, USA).

### 4.10. Cell Morphology Analysis by Fluorescence Microscopy

Cell morphology was analysed at the 24, 48, 72 and 96 h time points following the incubation with betulin colloidal particle dispersions at concentrations starting from 0.78 µg·mL^−1^ with a twofold increment up to 400 µg·mL^−1^ and subsequent fluorescent labelling of the cytoskeleton and nucleus. Following incubation with betulin dispersions, the cell culture medium was aspirated, and samples were washed twice with phosphate-buffered saline (PBS), fixed with a 4% formaldehyde diluted in PBS for 10 min and permeabilized in 0.1% Triton X-100 in PBS with 1% bovine serum albumin (BSA; all reagents were purchased from Sigma-Aldrich, St. Louis, MO, USA). The nuclei of the dermal fibroblasts were stained by Hoechst 33342 10 mg·mL^−1^ (Thermo Fisher Scientific, Rockford, IL, USA) diluted 1:1000 and the cell cytoskeleton was stained by ActinRed 555 (Thermo Fisher Scientific, Rockford, IL, USA) according to the manufacturers’ instructions. The samples were analysed using a TILL Photonics iMIC fluorescence microscope (TILL Photonics GmbH, Gräfelfing, Germany). Identical instrument settings were used for all samples. Exposure time was 100 ms. Images were processed using TILL Photonics Offline analysis software (TILL Photonics GmbH, Gräfelfing, Germany).

### 4.11. IL-8 and IL-1b Secretion Assessment by Enzyme-Linked Immunosorbent Assay (ELISA)

Cell culture supernatants were aspirated from cell cultures treated with betulin colloidal particle dispersions after 48 h and 96 h incubation, centrifuged and stored at −80 °C until further analysis. IL-8 secretion was assessed using DuoSet^®^ ELISA Human CXCL8/IL-8 (R&D Systems, Minnneapolis, MN, USA) and Human IL-1 beta ELISA (Sigma-Aldrich, St. Louis, MO, USA) kits according to the manufacturers’ instructions. Briefly, 96-well plates were coated with capture antibodies overnight. Cell culture supernatants were diluted twofold with reagent diluent 1% BSA in PBS, applied to 96-well plates coated with capture antibodies, and incubated for 2 h at room temperature (22 °C). Then supernatants were aspirated, and plates were washed three times with a wash buffer (0.05% Tween 20 in PBS). After the washing step, samples were incubated with biotinylated detection antibodies. After rigorous washing, a streptavidin-horseradish peroxidase conjugate was added to the plates. The washing procedure was repeated and 3,3′,5,5′-tetramethylbenzidine (TMB) substrate solution was applied and kept for 30 min in the dark. The reaction was stopped by 2N sulfuric acid, and the optical density was measured using a Tecan Infinite M200 Pro microplate reader and Magellan 7.1 SP1 software (Tecan Trading AG, Männedorf, Switzerland) at a wavelength of 450 nm.

### 4.12. Statistical Analysis

Statistical analysis was performed using GraphPad Prism Software (Graph Pad Inc., San Diego, CA, USA). The data used for analysis were representative results or the means of at least three independent experiments ± the standard error of the mean. Differences between studied groups were statistically assessed by ANOVA Tukey’s multiple comparisons test if not stated otherwise. Statistical significance is represented as * *p*-value < 0.05.

## 5. Conclusions

Our data show that after treatment of betulin with the antisolvent precipitation technique, an additional purification process (97 to 99 wt%) and morphological changes of the particles could be observed, resulting in a decrease in the average particle size in the aqueous dispersion from 552.9 ± 11.3 to 278.2 ± 1.6 nm. Obtained betulin colloidal particles showed no cytotoxicity up to a concentration of 400 µg/mL in the CCK-8 assay and induced the secretion of the proinflammatory and angiogenesis stimulating cytokine IL-8. Further research is needed to draw conclusions on betulin colloidal particle effect on skin dermal fibroblast function in wound healing.

## Figures and Tables

**Table 1:** Basic composition of the dry betulin samples.

Sample	Betulin [wt%]	Lupeol [wt%]	Betulinic Acid [wt%]	Phenolic Compounds [wt%]
Untreated betulin	97	0.4	1.2	0.28
Treated betulin	99	0.4	1.0	0.21

## Data Availability

The data presented in this study are available from the corresponding author on reasonable request.
